# The Impact of Structural and Functional Social Resources on Loneliness Among Americans Age 50 Years and Older

**DOI:** 10.1093/geroni/igab046.2070

**Published:** 2021-12-17

**Authors:** Jillian Minahan Zucchetto

**Affiliations:** Fordham University, United States

## Abstract

Social isolation and loneliness have many negative consequences (e.g., Cacioppo et al., 2006; Griffin et al., 2018; Uchino, 2006), especially among older adults (Perissinotto et al., 2012). According to the cognitive discrepancy theory (CDT), loneliness is the negative psychological state resulting from the perceived discrepancy between one’s desired level of social resources and one’s actual level of social resources (Peplau & Perlman, 1982; Perlman & Peplau, 1998). Social resources have both structural (e.g., objective) and functional (e.g., perceptions of the quality) aspects (Holt-Lunstad, 2017). The relationship between structural and functional social resources has been described as a filtration process in which functional aspects mediate the association between structural aspects and loneliness (Cacioppo et al., 2016; Hawkley et al., 2008, Hawkley & Kocherginsky, 2018). However, this filtration model has not been empirically tested within the CDT. This study examined the relationship among structural social resources (SSR), functional social resources (FSR), and loneliness cross-sectionally and longitudinally using a sample of 3,345 Americans aged 50 years and older from the 2008 and 2012 waves of the Health and Retirement Study. Results showed that there was a significant indirect effect both cross-sectionally (β = -.07) and longitudinally (β = -.06) such that FSR mediated the relationship between SSR and loneliness. Ultimately, the CDT is useful in explaining the complex relationship between structural and functional aspects of one’s social resources with loneliness, and interventions may seek to target the functional aspects of one’s social network to improve loneliness, rather than focusing solely on structural aspects.

